# A New Bioassay for the Detection of Paralytic and Amnesic Biotoxins Based on Motor Behavior Impairments of Zebrafish Larvae

**DOI:** 10.3390/ijms24087466

**Published:** 2023-04-18

**Authors:** Javiera F. De la Paz, Nicolás O. Zambrano, Fernando C. Ortiz, Alejandra Llanos-Rivera

**Affiliations:** 1 Laboratorio de Embriotoxicología e Interacción Desarrollo Ambiente (LEIDA), Departamento de Biología Celular, Facultad de Ciencias Biológicas, Universidad de Concepción, Concepción 4070386, Chile; 2 Laboratorio de Toxicología Acuática, Departamento de Oceanografía, Facultad de Ciencias Naturales y Oceanográficas, Universidad de Concepción, Concepción 4070386, Chile; 3 Danio Biotechnologies, SpA, Santiago 8271199, Chile; 4 Mechanisms of Myelin Formation and Repair Laboratory, Departamento de Biología, Facultad de Química y Biología, Universidad de Santiago de Chile, Av. Alameda 3363, Estación Central, Santiago 9170022, Chile

**Keywords:** zebrafish, bioassay, locomotor activity, biotoxin, saxitoxin, domoic acid, neurotoxin, HAB, harmful algal bloom, ZebraBioTox

## Abstract

The global concern about the increase of harmful algal bloom events and the possible impacts on food safety and aquatic ecosystems presents the necessity for the development of more accessible techniques for biotoxin detection for screening purposes. Considering the numerous advantages that zebrafish present as a biological model and particularly as a toxicants sentinel, we designed a sensitive and accessible test to determine the activity of paralytic and amnesic biotoxins using zebrafish larvae immersion. The ZebraBioTox bioassay is based on the automated recording of larval locomotor activity using an IR microbeam locomotion detector, and manual assessment of four complementary responses under a simple stereoscope: survival, periocular edema, body balance, and touch response. This 24 h acute static bioassay was set up in 96-well microplates using 5 dpf zebrafish larvae. For paralytic toxins, a significant decrease in locomotor activity and touch response of the larvae was detected, allowing a detection threshold of 0.1–0.2 µg/mL STXeq. In the case of the amnesic toxin the effect was reversed, detecting hyperactivity with a detection threshold of 10 µg/mL domoic acid. We propose that this assay might be used as a complementary tool for environmental safety monitoring.

## 1. Introduction

Zebrafish has been pointed out as a great model for the study of small molecules for the detection of toxicity and biological activities with therapeutic potential. It has been also used as an animal model for the study of drugs and mutations affecting basic responses such as locomotor activity or avoidance behavior, but also in the detection of more complex behaviors such as aggressiveness, anxiety, and addiction. Both simple and complex behaviors are controlled by the nervous system and light conditions [1]. Most of these studies use adult individuals; however, the use of zebrafish larvae for these purposes has increased in the last years, since larval stages present essential advantages for the study of substances that are expensive and/or difficult to obtain, such as natural extracts, toxins, or new drugs [2]. The small amounts of samples needed for the analysis reduces the cost, and the availability of automated detection systems for monitoring locomotor activity in real time allows the elimination of observation biases or variability due to the observer [3,4].

Zebrafish embryos are small, transparent, and present external fertilization, a high fecundity rate, and develop very rapidly and synchronously under controlled environmental conditions. Embryogenesis is complete only 72 h post-fertilization (hpf) at 28 °C, and in that period, hatching occurs, and a free-living larva is developed. In the next couple of days, most primary organ systems are functional, while yolk reserves are consumed. By 120 hpf, a functional nervous system has been developed, and the larva responds in a highly reproducible manner to physical stimulus and neuromodulator agents, showing easily quantifiable responses such as changes in the locomotor activity pattern, touch response, balance maintenance, and physiological manifestations of general toxicity such as edemas. Two neuromodulators that induce a nervous system depressive response in zebrafish larvae are the fish anesthetic Tricaine (3-aminobenzoic acid ethyl ester methanesulfonate, MS-222) and the paralytic biotoxin saxitoxin, STX [5,6,7].

Saxitoxins (STX) and gonyautoxins (GTX) are the main families of biotoxins present in the paralytic shellfish poison (PSP) produced by worldwide distributed marine dinoflagellates and freshwater cyanobacteria during harmful algal bloom (HAB) events [8]. PSP composition and toxicity are variable, but in general, PSP toxins, and especially STX, are highly dangerous to animals and humans that are accidentally exposed to contaminated food or water. STX produces fast and severe neurotoxic effects during acute poisoning, inducing a general paralysis that can result in respiratory arrest and death [9]. STX and its analogs are soluble and heat-stable trialkyl tetrahydropurines, known to act by binding with high affinity and blocking the voltage-gated ion channels in mammals and impeding the ingress of Na^+^ to the cell, but can also bind to voltage-gated K^+^ and Ca^2+^ channels [10,11].

Marine and freshwater harmful algal blooms (HABs)—including those producing PSP—are increasing globally due to climate change [12,13,14], putting food safety at risk by affecting fisheries and aquaculture, but also threatening drinking water supplies due to the increasing use of desalinization plants for drinking water production in coastal regions, and the pollution in freshwater sources, the availability of which is being increasingly reduced. Therefore, the risk of PSP and other biotoxins affecting human health is growing rapidly worldwide.

Public agencies monitor the presence of PSP biotoxins in foods constantly. The official protocols for PSP detection in bivalve shellfish meat include an analytical method based on chromatography with fluorescence detection, HPLC/FLD [15], and the mouse bioassay, MBA [16]. The HPLC/FLC is highly sensitive and precise, allowing for the detection of individual toxin concentrations and total PSP in the samples, but it requires very expensive equipment and specialized personnel, thus limiting the environmental and food safety monitoring. Moreover, this method does not give information about the global toxicity of complex samples, since it may not detect unknown PSP analogs or, depending on the PSP profile, the quantification of co-eluted analogs may not be possible [17]. On the other hand, MBA is fast, cheap, and sensitive enough for detecting samples at the regulatory limit (0.8 μg of Saxitoxin equivalents per gram (STXeq/g) in shellfish meat), showing a detection limit of 0.4 μg STXeq/g [18]. However, MBA is highly unspecific and presents bioethical issues due to the use of adult animals for experimentation. Indeed, although this bioassay is the official method in several countries, it is no longer used in the European Community countries [19]. In 2011, the group of Turner compared the analytical method based on HPLC/FLC with the MBA assay and reported that the latter can underestimate the results for some species of oysters [20]. Therefore, in the current global environment and fast population growth scenario, new accessible, rapid, and sensitive methods for the detection of these neurotoxins are still needed.

In this line, Lefebvre and cols. showed in 2004 that zebrafish embryos and larvae respond in a reversible and stage-specific manner to dissolved STX [5]. The investigators exposed the embryos to STX from 4 hpf to 7 dpf in 6-well plates, monitoring the individuals daily. They found several responses depending on the exposure duration: (i) morphological abnormalities (four to six days), (ii) the presence of periocular edemas (four days), (iii) a reduced touch response (two days), and (iv) paralysis (four days). In the latter case, the onset of the response was reduced in older larvae. Exposure to 0.37 μg STXeq/mL induced paralysis by 46 h on 2 days post-fertilization (dpf) embryos, and the same effect was observed by exposure times of eight and five hours in 4 dpf and 6 dpf larvae, respectively, with higher mortality rates in the latter cases [5]. The locomotor activity in adults and larval zebrafish is a simple behavior that has been reported to be affected by several drugs and environmental pollutants at sub-lethal concentrations [21,22,23,24,25,26] and is well known to be influenced by age, exposure volume, photoperiod, and group size [27,28,29].

A second kind of neurotoxin of interest in food security and environmental health is domoic acid (DA) and its isomers, small neurotoxic molecules produced by dinoflagellates of the *Pseudo-nitzchia* genus that compose the amnesic shellfish poison (ASP), which produces severe neurotoxic symptoms in humans that accidentally consume contaminated shellfish, suffering neurological damage that is virtually irreversible [30]. A regulatory limit of 20 μg/100 g was established after a massive intoxication event in 1987 in Canada, where more than 100 persons were affected and 4 of them died [31]. DA is an analog of L-glutamate, the principal excitatory neurotransmitter in the brain and spinal cord, and it is capable of binding to glutamate ionotropic receptors, stimulating these ion channels and consequently producing neuroexcitatory effects and neurodegenerative damage in animals, including fishes. An increase in the metabolic rate in the brain of Atlantic salmon after IP injection of 6 mg/Kg of DA [32] has been described, but developmental toxicity has also been observed after zebrafish eggs were injected, showing that the administration of DA in early life stages affects hatching, touch response, induces convulsions, an abnormally fast and constant pectoral fin movement in zebrafish [33], and negatively impacts on the neural development of zebrafish [34].

Considering all this information, the aim of this work was to develop a simple and accessible bioassay for PSP neurotoxin detection based on their global effect on zebrafish larvae. For this, we determined the most sensitive and easy-to-detect responses, the optimal stage of development and time of day to initiate the exposure, the minimum incubation volume, group sizes, the number of replicates needed, and the use of MS-222 anesthetic as a positive control. This standardization resulted in the obtention of a robust dataset that permitted us to evaluate several samples in parallel, in a sensitive and accessible semiautomated test. The designed bioassay was set up with a standard of pure STX, and tested with several STX analogs (NEO, dcSTX, GTX1&4, GTX2&3, and C1&2). Finally, we tested the effects of diluted domoic acid (DA) to evaluate the possibility of extending its utility to the detection of this amnesic neurotoxin by immersion.

## 2. Results and Discussion

### 2.1. Experimental Condition Setting

To create a bioassay protocol to expose zebrafish larvae to biotoxins by immersion using 96-well plates and measure locomotor activity (LMA) in real time, we used an automated detector system WMicrotracker^®^, which is based on infrared light scattering [35] and allows the recording of a small animal’s movement in real time. In order to define the optimal experimental conditions for this bioassay, we assess different environmental and experimental conditions to achieve the most reproducible results using the smallest amount of test samples or compounds, and larvae; therefore, the impact of the stage of development, incubation volume, group size, number of replicates, and time of day on the zebrafish LMA were evaluated under constant light conditions and using 30 min blocks of LMA recording for 2 to 6 h. Post-hatching larvae from four to seven days post-fertilization (dpf) were used for these experiments.

#### 2.1.1. Developmental Stage

**Figure 1:**
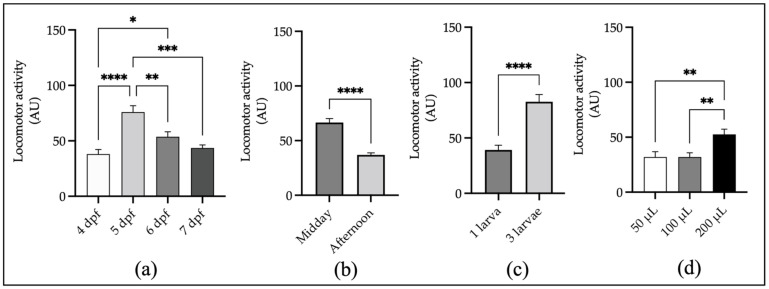
Experimental conditions. (**a**) *Developmental stage*: the automated activity registration of 4, 5, 6, and 7 dpf larvae for 2 h reveals that the 5 dpf stage larvae show the highest locomotor activity and that spontaneous motility decays with age (4 dpf, N = 78; 5 dpf, N = 72; 6 dpf, N = 78; and 7 dpf, N = 56). (**b**) *Time of day:* the LMA was found to be higher in the first half of the light phase of the photoperiod (11 am to 5 pm) in comparison with the second half (5 pm to 11 pm) in 5 dpf larvae (N = 50). (**c**) *Number of individuals per well:* the motility of individual 6 dpf larva presents lower LMA and dispersion of the data when compared with groups of 3 larvae per well (N = 30). (**d**) *Incubation volume:* the average LMA of 6 dpf larvae was not significantly different when registered in 50 μL to 100 μL, but activity was significantly higher when incubated in 200 µL per well (N = 30). The data from four independent experiments were pooled for (**a**,**b**), three experiments for (**c**), and two experiments for (**d**). Mean ± SE. AU, arbitrary units of locomotor activity registered as the number of reads/well in 30 min blocks (bin30). * *p* < 0.05; ** *p* < 0.01; *** *p* < 0.001; **** *p* < 0.0001. N = number of individuals.

Our results show a clear difference in the larval LMA at different stages of development. The 4 dpf larvae showed little motility on average and high variability between individuals, which is reflected by a high standard deviation (SD), standard error (SE), and coefficient of variation (CV) of the mean (M) recorded motility data in arbitrary units AU (M: 38.1; SD: 35.6; CV: 93.4%; and N = 78). This is probably because at this stage the swim bladder is air-inflated in most but not all individuals, and the nervous system is still developing. By contrast, 5 dpf larvae show the highest motility and lower variability (M: 75.9; SD: 49.0; CV: 64.6; and N = 72) compared with the LMA at 7 dpf (M: 43.5; SD: 21.1; CV: 48.6; and N = 56) (Figure 1a), probably due to the depletion of yolk nutritional reserves at this stage. These results are consistent with previous reports using other LMA recording systems based on video tracking [36,37,38].

#### 2.1.2. Time of Day

When comparing the motility of 5 dpf larvae during the daytime to midnight under constant light conditions starting 3 h after the light phase of the 14 h light/10 h dark photoperiod, we detected the highest average activity during “midday” between 11 am to 5 pm (M: 66.6; SD: 25.9; CV: 38.9; and N = 50), while the LMA decreases during the “afternoon” between 5 pm to 11 pm (M: 36.9; SD: 13.3; CV: 36.0; and N = 50) as shown in Figure 1b, this is also in agreement with previously reported data [29].

#### 2.1.3. Group Size and Incubation Volume

We also found that data dispersion (Figure 1c), measured as SD and SE of the mean, is lower when one individual per well is used (M: 39.2; SD: 23.0; SE: 4.2; CV: 58.5; and N = 30) compared with three individuals per well (M: 82.7; SD: 35.8; SE: 6.5; CV: 43.3; and N = 30). Other authors have argued that data dispersion is smaller when three individuals per well are used [39]. In our hands, this premise is verified only when we considered the CV as a measure of variability. On the other hand, the LMA of 5 dpf individuals is not affected by small incubation volume ranging from 50 to 100 μL (Figure 1d), but it is significantly higher when the incubation volume is 200 μL. This is important since a small incubation volume allows the testing by immersion of expensive compounds, as is the case of marine biotoxin standards, difficult to obtain extracts such as the fish egg’s perivitelline fluid [40] or others, using small amounts of test substances or samples without compromising the reproducibility of the results and the robustness of the statistical analysis by the reduction of technical replicates.

### 2.2. Replicate Number and Controls

In order to determine the smallest number of replicates necessary to obtain reproducible and statistically robust results, we calculate the minimal number of replicates needed as described in the Materials and Methods. The test result shows that the ideal number of replicates (the number of wells per treatment group using one larva per well in 50–100 μL incubation volume), is equal to eleven. Therefore, in each 96-well plate, eight treatment groups or samples can be evaluated simultaneously in order to achieve the lowest limit of detection (LOD) possible with this protocol for the evaluation of paralytic neurotoxins, and other nervous system depressants.

### 2.3. Kinetics of Locomotor Activity in Zebrafish Larvae Exposed to Saxitoxin (STX)

After determining the optimal experimental conditions for LMA detection with zebrafish larvae using the automated recording system, first we evaluate the kinetic of the impact of solved STX on larvae motility and the minimum concentration of this neurotoxin that induces a significant effect during a period of time, and therefore the optimal duration and limit of detection (LOD) of the method. STX pure standard was dissolved in an E3 culture medium, and the motility of larvae was compared with a negative control (E3), a solvent control (HCl 0.05 mM) equivalent to the highest STX dilution (0.8 µg/mL STX), and a positive control (Tricaine 0.002%) that was previously determined as the lowest concentration that significantly reduces the LMA without total paralysis.

**Figure 2:**
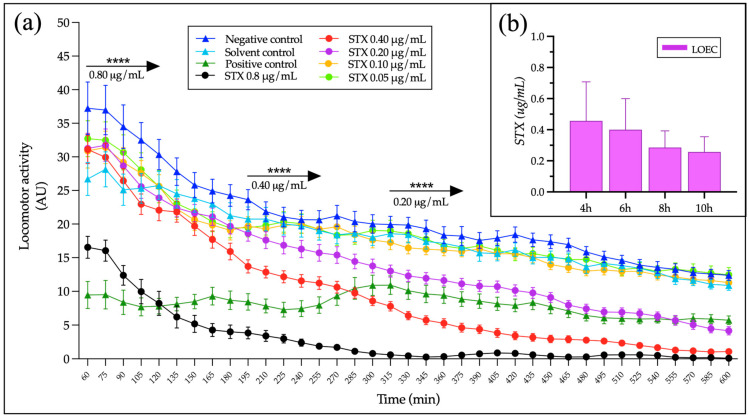
STX effect on zebrafish larvae locomotor activity (LMA). The pooled data from six independent experiments are presented. (**a**) Kinetics of STX effect at different concentrations on 5 dpf zebrafish larvae; the left end of each arrow indicates the time point when the effect starts to be significant for each concentration according to the statistical analysis. The lowest STX concentration that can be automatically detected with this method was equal to 0.2 µg/mL in five of seven experiments performed. AU: movements registered on 15 min intervals presented as a moving average per hour ± SE. Negative control, N = 60; solvent control, N = 60; positive control, N = 54; STX 0.80 µg/mL, N = 36; STX 0.40–0.10 µg/mL, N = 70; and STX 0.05 µg/mL, N = 59. (**b**) The average LOEC on zebrafish LMA exposed to STX over exposure time is shown. Note the tendency (not significant) of a reduced LOEC as exposure time increases. After 10 h of exposure, a concentration of ~0.25 µg/mL was detected. Mean ± SD.

Our results demonstrated that STX decreases the LMA in a concentration-dependent fashion and as expected, at higher concentrations the effect is faster. Exposure to 0.8 µg/mL of STX induces a decrease in LMA that can be detected in an hour, half of this concentration can be detected after 3.25 h of exposure, and the impact of 0.2 µg/mL STX is significant after 5 h (Figure 2a). In order to determine the LOD for our test, the data of six independent 10 h experiments using serial dilutions of STX from 0.8 µg/mL to 0.05 µg/mL in the E3 medium, and performed by two different researchers was analyzed, and the lowest effect concentration (LOEC) for different exposure (registration) times was determined for each experiment. Although we found a tendency of a decreased LOEC with longer time of exposure (Figure 2b) this correlation was not statistically significant. However, this analysis allows us to find an average LOEC as low as 0.26 µg/mL of STX after 10 h of exposure time (IC-95%: 0.17–0.35; SD: 0.098; SE: 0.037; and CV: 38.0%) showing a CV = 38% compared to values over CV = 50% in shorter exposure times. Thus, our results indicate that running the trials by using 10 h of exposure time reduces the data variability and permits the detection of values as low as 0.26 μg/mL, at least for STX. According to this result, the duration of the exposure to maximize the test sensitivity was set at ten hours, with a minimum LMA registration of four hours between six and ten hours of exposure under constant light conditions. If the registration time is reduced to two hours in this range of time, the LOEC increases to 0.4 µg/mL of STX, a sensitivity that is enough to detect STX levels above the equivalent limit established for food safety purposes, equal to 0.8 μg/g.

### 2.4. Complementary Responses of Zebrafish Larvae to STX

**Figure 3:**
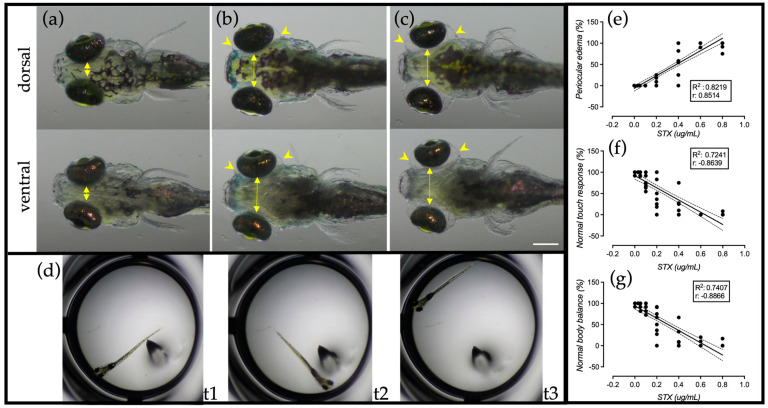
Complementary responses to STX exposure. Zebrafish larvae were exposed for 24 h to (**a**) E3 medium negative control; (**b**) STX 0.2 µg/mL; and (**c**) STX 0.4 µg/mL. STX induces the occurrence of periocular edemas (arrowheads) that are evident due to the increase of eye separation (yellow arrows) when the larva is seen from a dorsal or ventral view; the normal dorsal position that reflects the maintenance of body balance is easily detected by identifying the dorsal stripe of pigmentary cells in the head region. (**d**) The touch response can be assessed by touching the tail of the larva with a fine needle as shown in t1, and a normal response is registered when the larva moves away from the stimulus source as shown in t2 and t3. (**e**–**g**) Simple linear regression for sub-lethal CRs. Scale bar: 200 µm.

To evaluate other effects or complementary responses (CRs) that can indicate the presence of STX and other neurotoxins, after 24 h of exposure the individuals from the previous experiments were evaluated for the presence of other signs of toxicity under the stereoscope: the heartbeat to assess survival, the occurrence of periocular edemas, the touch response—responses that were previously described by Lefebvre et al. (2004) [5] after STX exposure—and the capacity of larvae to maintain its normal body balance were registered (Figure 3). The results summarized in Table 1 revealed that acute exposure to STX up to 1.2 µg/mL is not lethal to zebrafish larvae at this stage but produces total paralysis and periocular edemas in >90% of the individuals, while larvae exposed to 0.03 to 0.05 µg/mL of STX did not present any sign of toxicity using this method. On the other hand, 0.1 µg/mL of STX and above induced an abnormal touch response and loss of body balance in >10% of individuals. Exposure to 0.2 µg/mL STX and above induced the formation of periocular edemas (~19%) and paralysis in approximately half of individuals, evidenced by the total loss of touch response (~54%) and the capacity of maintaining body balance (~51%). Paralysis is total but not lethal to individuals exposed to 0.8–1.6 µg/mL of STX, while exposure to 2.2 µg/mL STX is lethal to ~25 of the test individuals. The touch response has been previously used by several authors to assess genetic mutations [36] or chemical compounds producing neuromotor disturbance in zebrafish larvae, as the impact of prolonged STX exposure in early developmental stages in zebrafish and Pacific herring [5,41]. Granato et al. (1996) assigned categories to this natural escape behavior according to the traveled distance away from the stimulus source [36], but in our bioassay where larvae are confined in a small space, the traveled distance cannot be evaluated due to the small size of the well, and therefore it is not possible to assign those categories to the touch response; however, according to our results, a “simple” dichotomic touch response presents a good correlation with the STX concentration (r = 0.8639 and R^2^: 0.7241), as do the normal body balance (r = 0.8866 and R^2^: 0.7407) and periocular edemas (r = 0.8514 and R^2^: 0.8219), as shown in Figure 3e–g.

The high correlation and sensitivity of the CRs evaluated represent a confirmation of the robustness of the semiautomated test designed. They allow us to suggest that the determined LOD of 0.2 to 0.4 µg/mL of STX using the LMA could even be reduced to 0.1 to 0.2 µg/mL of STX if these complementary responses are considered as a confirmatory and complementary part of the test, and to project that it could be possible to estimate concentration ranges for STX and other PSP toxins based on the level and combination of LMA and these responses in future work.

According to these results (Table 1), the lowest STX concentration tested that produced a statistically significative reduction in zebrafish larvae LMA (0.1–0.2 µg/mL), in ten hours of exposure, also induces an abnormal touch response and loss of body balance in more than 10% of the individuals (Table 1) after 24 h of exposure, while negative and solvent control groups presented these complementary responses in less than 1% and 3% of the individuals, respectively. Therefore, based on the results obtained from several experiments, we considered that any response that is presented in ≥10% of the test individuals in the bioassay is positive for motor toxicity, and we established that the ZebraBioTox assay was positive for the presence of STX when (a) the LMA is significantly reduced, and/or (b) two complementary responses are presented in ≥10% of test individuals. If by chance the negative control presents any response in at least 10% of individuals, the assay is invalidated, since this could be a sign of an abnormal sensitivity in the batch of larvae caused by external or maternal factors (culture media contamination, reduced quality of the eggs, etc.), and the results of the test would not be reliable.

Since other pollutants can affect LMA in zebrafish, the use of this CR that—as mentioned above—has been previously described after STX exposure in zebrafish, increases the specificity of the assay.

### 2.5. Determination of the Relative Toxic Potency of Other PSP Biotoxins Using the ZebraBioTox Bioassay

**Figure 4:**
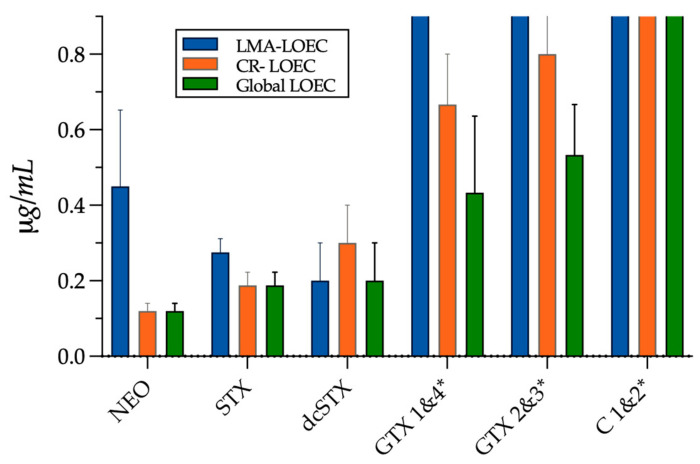
ZebraBioTox bioassay applied to several PSP biotoxins. The average LOEC ± SE for STX and its analogs, NEO, dcSTX, GTX2&3, GTX1&4, and C1&2, was determined by the evaluation of the LMA (blue bars), the complementary responses (orange bars), and the global test result (green bars) using in each case the lowest value given by the LMA and/or the CRs. * Note that there were no responses to C1&2 in any tested concentration, and no effect on LMA was detected for GTX1&4 and GTX2&3 in at least one of the experiments pooled (LOEC ≥ 0.8 µg/mL).

It is well known that PSP presents a variable composition, and its toxicity depends on the presence and relative concentration of different STX analogs. Therefore, to further evaluate the ZebraBioTox bioassay usefulness for PSP detection we tested serial dilutions of other neurotoxins of the STX family, NEO, dcSTX, GTX2&3, GTX1&4, and C1&2, to determine the LOEC value for each of them by considering the LMA and the CRs alone, and the combination of both parts of the assay to obtain a global LOEC (Figure 4). Later, we compared the relative toxicity of each STX analog with the relative potency reported for these toxins using other biological PSP detection methods based on mammalian models: the MBA and the inhibition of sodium channels in cultured neural cells from mice (Table 2).

The result of this exercise reveals that not all the STX analogs induce the same responses in the zebrafish larvae as STX, and therefore the use of these complementary responses is a key step of the bioassay to validate its sensitivity and specificity, but at the same time, the CRs are not sufficient to determine toxin effects. For example, STX and dcSTX present a very similar LOEC for LMA and CRs (~0.2–0.3 µg/mL), while for NEO the concentration that significantly reduces the LMA (0.5 µg/mL) is five times higher than the concentration needed to affect CRs (0.1 µg/mL). On the other hand, we found that the most sensitive response for all the PSP analogs is the touch response, closely followed by the loss of body balance, and even when periocular edemas are present in the LOEC concentration for NEO and STX, they are absent in the equivalent concentrations of dcSTX, GTX1&4, GTX2&3, and C1&2, and only appear in individuals exposed to the medium to high concentration (>LOEC) of those used in our experiments.

Later, we compared the relative toxicity of PSP neurotoxins obtained with the relative toxicity reported by other authors using different biological methods, as shown in Table 2. We found that the relative toxicity of PSP analogs to zebrafish larvae is very similar to what is reported for mice using the MBA test [42,43], which presents a limit of detection of 0.4 µg/mL eqSTX and it still is the official method for PSP detection in several countries, and for in vitro VGSC inhibition on neural cell cultures [44], suggesting that the impact of these PSP neurotoxins on zebrafish larvae is similar to what is observed in mammals.

**Table 2:** Relative toxicity of PSP biotoxins detected by ZebraBioTox. The obtained results from three independent experiments for each toxin standard allowed us to determine their relative toxicity for zebrafish larvae. A comparison with the relative toxicity reported for other biological methods is presented.

Order of Potency ^1^	ZebraBioTox ^2^	MBA ^3^ [43]	In Vitro ^4^ [44]	MBA ^3^ [42]
1°	NEO	STX	NEO	STX
2°	STX-dcSTX	NEO-dcSTX	dcSTX-STX	NEO
3°	GTX1&4	GTX1&4	GTX1&4	GTX1–GTX4
4°	GTX2&3	GTX2&3	GTX2&3	dcSTX
5°	C1&2	-	C1&2	GTX2–GTX3

^1^ The order of potency for PSP biotoxins according to each method from the most to the least toxic; ^2^ ZebraBioTox global assay sensitivity (considering both LMA and CRs) using larvae immersion; ^3^ MBA, mouse bioassay using intraperitoneal injection; ^4^ in vitro test for the inhibition of neuronal sodium channels of mice cerebellar granular cells.

### 2.6. Zebrafish Larvae Responses to Domoic Acid (DA) Exposure

**Figure 5:**
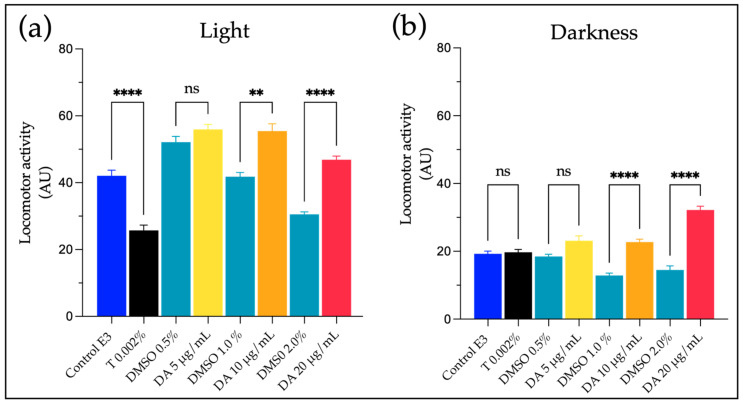
Domoic acid induces hyperactivity in zebrafish larvae. The exposure of zebrafish larvae to DA through immersion is capable of inducing a significant increase in the LMA that can be detected in (**a**) light and, (**b**) darkness conditions, although in the latter one the effect is more notorious. AU: movements registered on 15 min intervals during 7 h (light) and 10 h (dark). Mean ± SE; N = 12–48; ** *p* < 0.01; **** *p* < 0.0001, ns: not significant.

To evaluate if ZebraBioTox bioassay use could be extended to DA as a representative of other types of neurotoxins that present different mechanisms of action than PSP, we applied the bioassay to determine if this toxin could also affect zebrafish larvae after exposure through immersion, an administration route that is more ecologically relevant than injection. The exposure to dissolved DA induces different responses in zebrafish larvae than STX, increasing the LMA and producing significant hyperactivity, consistent with an agonist of the glutamate receptors such as DA. Since all the experimental conditions for LMA testing in our assay were adjusted to increase the sensitivity for detection of paralytic neurotoxins, we evaluated the impact of DA not only in constant light conditions during midday (Figure 5a), but also during the low activity phase of the circadian rhythm of larvae and in constant darkness (Figure 5b), and we found that 10 µg/mL of solved DA can induce hyperactivity and that this effect is more significant when evaluated in darkness. When the CRs were evaluated, we found that DA affects the normal touch response after exposure to >5 µg/mL of DA (M: 84.7; SD: 4.03; and N = 24), and the loss of body balance occurs when concentrations equal to and above 15 µg/mL are used (M: 65.0; SD: 49.5; and N = 24), and no signs of periocular edemas were detected at any concentration tested (2.5 to 20 µg/mL), demonstrating that the use of CRs increases the specificity of our bioassay and could possibly allow discriminating the kind of biotoxins present in environmental samples, although more studies are needed to confirm this.

Our results showed that the exposure of zebrafish larvae to DA through immersion is useful to evaluate its impact and potentially its detection in liquid samples.

Altogether, these results show that the ZebraBioTox bioassay is sensitive enough to be used for neurotoxin detection in liquid samples, in a simple and cost-efficient test that does not require highly expensive or complex equipment, complicated calculations, or highly trained personnel. Since the results are based on complex responses of the whole organism, the test allows the detection of the presence of toxic levels of PSP, something that the analytical methods cannot do directly.

The results obtained from experiments with STX, its analogs, and DA showed that the sensitivity of the complementary responses in decreasing order is: *abnormal touch response ≥ loss of body balance > periocular edemas > survival.* The method allows the detection of low PSP concentrations of 0.1–0.2 μg/mL STXeq.

Improvements to our method will possibly allow us to obtain semi-quantitative results and to discriminate between different types of biotoxins. Therefore, our results allow us to project the use of the ZebraBioTox bioassay as a complementary test to the official methods, but for this, the assay of different combinations of PSP biotoxins, among others, and interlaboratory studies should be conducted in the future.

## 3. Materials and Methods

### 3.1. Animals

Zebrafish adult wild-type strains were maintained and raised in our facility under recommended conditions [45] and fed twice a day using GEMMA micro 500 Skretting (Westbrook, ME, USA). The fish strains used, AB and *TAB5*, were a kind gift from Miguel Allende (Universidad de Chile). Embryos were obtained in spawning boxes; 60 min after the light was turned on, eggs were collected and rinsed using E3 embryo medium (5 mM NaCl, 0.17 mM KCl, 0.33 mM CaCl2, and 0.33 mM MgSO4, equilibrated to pH 7.2) and maintained at 28 ± 0.5 °C in Petri dishes, at a density of no more than 60 embryos per dish, and cleaned daily. Dead and abnormal individuals were removed immediately. Egg batches with viability <80% approximately, were discarded. Embryonic and larval ages are expressed in hours post-fertilization (hpf) or days post-fertilization (dpf). All procedures comply with the guidelines of the Animal Ethics Committee of the Universidad de Concepción.

### 3.2. Chemical and Reagents

All the toxins used in this study were certified reference materials purchased from the Institute for Marine Biosciences (National Research Council Canada, Halifax, NS, Canada) and were maintained at 4 °C or −20 °C according to the safety data sheets (CMR-STX-f Lot#20110316; CRM-DA-g Lot#20140730; CRM-dcSTX-b Lot#20110713; CRM-NEO-d Lot# 20170411; CMR-GTX2&3-d Lot#20170419; CMR-GTX1&4-d Lot#20160608; and CRM-C1&2-b Lot#20110706). Tricaine (MS-222), chlorohydric acid (HCl), and acetic acid were purchased from Sigma-Aldrich (St. Louis, MO, USA), and dimethyl sulfoxide (DMSO) from Merk (Darmstadt, Germany), and were diluted separately in distilled water.

### 3.3. Exposure Solutions and Experimental Groups

Experimental solutions ranging from 0.8 µg/mL to 0.1 µg/mL for PSP and 2.5 µg/mL to 20 µg/mL for ASP, were obtained by diluting the standard toxins with filtered, autoclaved E3 medium. In the case of DA, the solvent acetonitrile 5% from an aliquot of the standard was evaporated using N_2_ flow injection in a glass vial in a thermoblock at 50 °C, and the toxin was resuspended in DMSO 2% before being diluted in E3. The treatment groups included a negative (E3 medium), positive (Tricaine at 0.002%), and blank group (E3 medium without larva) as internal controls, and the exposure groups included a solvent control at the concentration in which each toxin at the highest concentration was solved: HCl 0.05 mM for PSP toxins and DMSO 2% for DA and the groups exposed to the dilutions of each toxin.

### 3.4. Locomotor Activity Measurement

Five dpf larvae with normal touch response, a fully filled swim bladder, and without any type of morphological or behavioral abnormality were collected and randomly distributed into a 96-well plate with one larva per well, containing 100 μL of each treatment solution (unless otherwise stated); for this operation, we cut off the end of a p200 tip to avoid damaging the larvae and maintain a controlled volume of incubation. An acclimatization period of 30 min in 50 μL of volume was used, and subsequently, test solutions at a 2× concentration were added to each well using a multichannel pipette just before LMA recording started; therefore, the recording and incubation time are equivalent in the graphs. Eleven replicate wells were used for each treatment group. The locomotor activity, defined as spontaneous larvae movement, was quantified under constant light conditions for 10 h in an infrared (IR) microbeam scattering detector (WMicrotracker ONE, PhylumTech, Santa Fe, Argentina). To improve the assessment of this parameter we used a 15 min period (bin size) following Bichara et al. (2013) [35], unless otherwise stated.

### 3.5. Complementary Response Assessment

After 24 h of exposure, four complementary responses were evaluated: (1) survival rate, (2) body balance, (3) touch response, and (4) the presence of periocular edema, were assessed under a stereoscopic microscope (Carl Zeiss, Jena, Germany) and registered.

#### 3.5.1. Body Balance

This is the first CR to be evaluated. The buoyancy position of an animal is determined, without touching it. If the animal is in a relatively horizontal position and with its dorsal side facing up (Figure 3a), the position is normal; on the contrary, the animal is found with its lateral or ventral side facing up (Figure 3b).

#### 3.5.2. Periocular Edema

The presence of periocular edema is easy to observe in animals in a ventral or dorsal position. It is evidenced by an increase in the distance between the animal’s eyes and the accumulation of fluid around the eyeball (Figure 3b).

#### 3.5.3. Touch Response

To assess the normal touch response, the dorsal side of the animal’s trunk or tail is touched with a fine pin a maximum of three times. If the animal moves away from the stimulus, the response is considered normal.

#### 3.5.4. Survival

Survival is assessed by the detection of a heartbeat, defined as a systolic contraction of the myocardium. Due to the transparency of the zebrafish embryo, this contraction can be visualized directly using a stereoscope, after manipulation of the larvae using a fine plastic tip to observe their ventral or lateral side. This is the last response to be evaluated in paralyzed animals.

### 3.6. Data Processing and Statistical Analysis

Statistical analyses were performed using GraphPad 9 (GraphPad Software, La Jolla, CA, USA). Experimental results are expressed as the mean ± the standard error (SE) or the standard deviation SD of the mean. Differences were considered statistically significant at *p* < 0.05. The data structure was tested for normality using the D’Agostino and Pearson test before comparison with the proper statistical test.

#### 3.6.1. Experimental Conditions Data Analysis

Statistical analysis of the developmental stage and incubation volume data was performed by using the Kruskal–Wallis test (Dunn’s multiple comparison post hoc). For the time of the day and group size evaluation, the two-tailed Mann–Whitney test was used.

#### 3.6.2. LMA Data Analysis

Statistical differences between treatment groups in LMA quantification were carried out using two different approaches: (a) The moving hourly average for the row data was calculated, and then the multiple Mann–Whitney test with multiple comparisons by the Holm–Šídák method was used to determine the time points of significant effects. (b) To compare the LMA of groups regarding time, the Kruskal–Wallis test (Dunn’s multiple comparison post hoc) was used.

#### 3.6.3. CR Correlation Analysis

To determine the relationship between STX concentration and each of the sub-lethal CRs assessed, the goodness of fit (R^2^) was estimated from a simple linear regression and the Spearman correlation coefficient (r) was calculated using a 95% confidence interval level.

#### 3.6.4. Determination of the Minimum Number of Replicates

The number of replicates was calculated by applying the “F test” to the pooled data of several independent experiments on 5 dpf larvae with STX dilutions and the experimental conditions previously determined, using the G Power software (version 3.1.9.7). Standard alpha and beta values of 0.005 and 0.2, respectively were set, and only the LMA was used as a predictor parameter. The “Effect size” was calculated based on the minimum significative correlation coefficient of (0.5) between the response (LMA) and the toxin exposure (STX concentration) near the lowest concentration with an observed effect (0.1 µg/mL STX). It is important to mention that this correlation value (R^2^) increases significantly in the higher toxin sub-lethal concentrations that were evaluated (0.4 and 0.8 µg/mL STX).

## 4. Conclusions

In this work, we set up a bioassay for the detection of dissolved PSP and DA biotoxins. We determined the ideal experimental and environmental conditions to achieve the lowest limit of detection possible in an acute bioassay of only 24 h. Our result revealed that 5 dpf zebrafish larvae present the highest locomotor activity, reaching a maximum activity during midday, and that the data dispersion is reduced when larvae are incubated individually in a small volume (50–100 μL) using 96-well plates. We also determined that a reduced number of test individuals (eleven per treatment group) is enough to obtain highly reproducible results for the detection of locomotor behavioral impairments produced by the exposure of zebrafish larvae to natural neurotoxins by immersion.

Altogether, our results show that the ZebraBioTox bioassay is a simple, accessible, and highly sensitive tool for the detection of neurotoxic compounds and molecules, such as those present in PSP and ASP, and that it can be potentially used as a complementary tool for environmental monitoring in water and food safety programs since it presents enough sensibility for the detection and discrimination of the PSP and ASP biotoxins below the regulatory limits of these groups of biotoxins.

## Figures and Tables

**Table 1:** Descriptive statistics of complementary responses (CRs) to STX exposure. The pooled data from several independent experiments are presented. The mean value ± SD for each CR is presented. Values >10% are considered a positive response and highlighted in bold. The number of experiments pooled (eleven larva/experiment) is shown in parentheses.

**CR (%)**	**Control (-)**		**Solvent**		**STX 2.2 µg/mL**		**STX 1.6 µg/mL**		**STX 1.2 µg/mL**		**STX 0.8 µg/mL**		**STX 0.4 µg/mL**		**STX 0.2 µg/mL**		**STX 0.1 µg/mL**	
**Mortality**	0.0 ± 0	(20)	0.0 ± 0	(10)	**25.0** ± 17.7	(2)	9.7 ± 8.7	(3)	0.0 ± 0	(4)	2.8 ± 4.3	(6)	0.0 ± 0	(10)	0.0 ± 0	(9)	0.0 ± 0	(9)
**Periocular edema**	0.0 ± 0	(20)	0.0 ± 0	(10)	**100** ± 0	(2)	**100** ± 0	(3)	**100** ± 0	(4)	**94.4** ± 10.1	(6)	**66.0** ± 33.8	(10)	**18.9** ± 31.9	(9)	0.0 ± 0	(9)
**Abnormal touch response**	0.8 ± 2.6	(20)	1.7 ± 5.3	(10)	**100** ± 0	(2)	**100** ± 0	(3)	**100** ± 0	(4)	**98.6** ± 3.4	(6)	**83.8** ± 23.8	(10)	**54.2** ± 39.9	(9)	**20.5** ± 16.8	(9)
**Loss of body balance**	0.8 ± 2.6	(20)	2.5 ± 5.6	(10)	**100** ± 0	(2)	**100** ± 0	(3)	**100** ± 0	(4)	**97.2** ± 6.8	(6)	**88.3** ± 21.9	(10)	**51.3** ± 35.4	(9)	**11.7** ± 10.3	(9)

## Data Availability

Not applicable.
